# Setting Criterion for Adolescent Circuit Exercise Program

**DOI:** 10.3390/ijerph18199996

**Published:** 2021-09-23

**Authors:** Yeon-Oh Han, Byung-Sun Lee, Seon-Yeong Shin

**Affiliations:** 1 WEPEAK, Subin Art Inn Building, 25 Eonju-ro 159-Gil, Gangnam-gu, Seoul 06024, Korea; 2 Health Physical Activity Institute, Subin Art Inn Building, 25 Eonju-ro 159 Gil, Gangnam-gu, Seoul 06024, Korea; shotace@khu.ac.kr (B.-S.L.); healthlab.shin@gmail.com (S.-Y.S.)

**Keywords:** adolescents, physical education, physical activity, physical fitness, circuit exercise program, criteria

## Abstract

The purpose of this study was to develop criteria for an adolescent circuit exercise program. The subjects of this study were 5268 middle- and high-school students. It consisted of three types of circuit exercise programs which were conducted in the physical education class. In the result of this study, we have found two significant finding. First, there were statistically significant differences by grade level and gender in three types of circuit exercise programs. Second, in order to improve the utilization rate and convenience of various adolescents’ physical activity environments and the field of school physical education, the gender of each circuit exercise program was classified and the five-grade evaluation criteria were presented. The criteria for circuit exercise program developed in this study will be utilized for various youth physical activities to contribute to improving health and physical fitness. In addition, physical education teachers are expected to use this criteria as a standard for evaluating the physical fitness level of adolescents.

## 1. Introduction

The decline of physical activity in adolescents is emerging as a global concern. According to the WHO’s survey of adolescent physical activity in 2020, four out of five adolescents worldwide were found to lack physical activity. In particular, 94.2% of Korean adolescents reported a significant lack of physical activity [1]. Excessive physical activity can have a negative effect on adolescents, but an appropriate level of physical activity has a positive effect on mental and physical health. In addition, physical activity habits in adolescence appear to be health levels and physical activity habits in adulthood after growth. Physical activity for adult health levels is encouraged to begin as early as adolescence [2]. Various guidelines are being provided to create healthy physical activity habits for adolescents. The World Health Organization (WHO) recommends that moderate to vigorous physical activity (MVPA) be carried out for more than 60 min each day, considering the development of adolescents’ physical and mental health and the effects on their entire lives [3]. The guidelines for physical activities of children and adolescents provided by the Ministry of Health and Welfare also recommends MVPA (aerobic exercise) of more than 60 min per day and strength exercise of more than 60 min per week [4].

The importance of school physical education classes is emphasized as an important means of maintaining adolescents’ physical activity habits and fitness levels. During vacations without physical education classes, students had the lowest level of MVPA, and during the semester, the day without physical education was lower than the day when physical education classes were held. Therefore, school physical education classes have a significant impact on the MVPA of adolescent students [5]. Six out of 10 Korean adolescent students do not participate in physical activities other than physical education classes held in schools. As a result, adolescent physical fitness is decreasing while their physique is growing [6]. In addition, the number of physical education classes is decreasing, suggesting the need for various efforts to improve the physical fitness and health of adolescent. The school’s physical education site is conducting a physical activity promotion system (PAPS) as a way to prevent obesity and physical fitness deterioration of students. The PAPS provides information on the health and physical fitness levels of adolescent students by presenting a five-step criteria [7]. Various efforts are being made to improve health and physical fitness for students who are at low levels in the PAPS. However, the lack of motivation, feedback, and exercise prescriptions has left adolescents unable to improve their physical fitness [8]. Students lack interest in physical activity programs and lack the willingness to participate in activities unrelated to grades or college entrance. Teachers are experiencing practical difficulties in guidance due to a lack of health and physical fitness programs. There is a need to find adolescents’ health and physical fitness programs that can provide students’ interests and teachers’ convenience [9].

Circuit exercise programs (CEP) are a form of exercise program that carries out a variety of exercise movements in a fixed order. In previous studies, the CEP was reported as an effective exercise method to improve the health and physical fitness of adolescents [10,11]. A study by Moyorga-Vega et al. (2013) found that the CEP was effective in improving students’ physical fitness at school physical education sites. It was reported as an effective exercise method that allows participation in various forms of exercise with less exercise time [12]. The Healthy Physical Activity Institute has developed a circuit exercise program called the “Move Challenge” consisting of various fitness movements. The Move Challenge has been proposed to strengthen health and physical activity management capabilities by satisfying the amount of physical activity recommended for students, and to help students perform safe and effective physical exercise [13]. However, the link between the evaluation criteria of physical fitness level and the operation of health and physical fitness improvement programs through physical activity practice is insufficient. The establishment of absolute fitness evaluation criteria for adolescents may provide specific and individual diagnostic information regarding the compliance of health and physical fitness. On the other hand, the need for appropriate evaluation criteria has been suggested for physical activity programs, as the criteria may be arbitrary and the disadvantages of classification errors may arise [14]. Criteria are needed to evaluate the results of adolescents’ health and physical fitness programs conducted on-site in physical education and to confirm the degree of improvement.

This study aims to present the evaluation criteria for the adolescents’ CEP constructed using fitness movements. CEP is effective in improving health and physical fitness. It will provide teachers with convenience in physical education classes. In addition, voluntary participation will be possible by presenting goals for each physical level to students. Therefore, by developing the evaluation criteria of the adolescents’ CEP, we intend to provide a physical activity program that is linked to the adolescent’s health and physical fitness improvement and evaluation of physical fitness level.

## 2. Materials and Methods

### 2.1. Subjects

The subjects of this study were adolescents aged 14 to 19 (first grade to sixth grade) in about 200 middle and high schools. It was conducted for students who expressed their intention to participate in CEP in school physical education classes. If a record was omitted among the CEP results, it was excluded from each CEP result analysis. In addition, if the institution or subject did not want, they were excluded from the subject. The physical education teacher fully explained the purpose and method of this study and was provided with physical activity (CEP) records. Physical activity data of 5268 people (male: 3082, female: 2186) were analyzed. In order to secure the homogeneity of the circuit exercise program records, teachers at the recruited schools were fully trained on how to implement the circuit exercise program through teacher training. All subjects’ data are secondary data, and only physical activity records excluding personal information is handled. The physical characteristics of the subjects are shown in Table 1.

### 2.2. Method of Circuit Exercise Program

**Figure A1:**
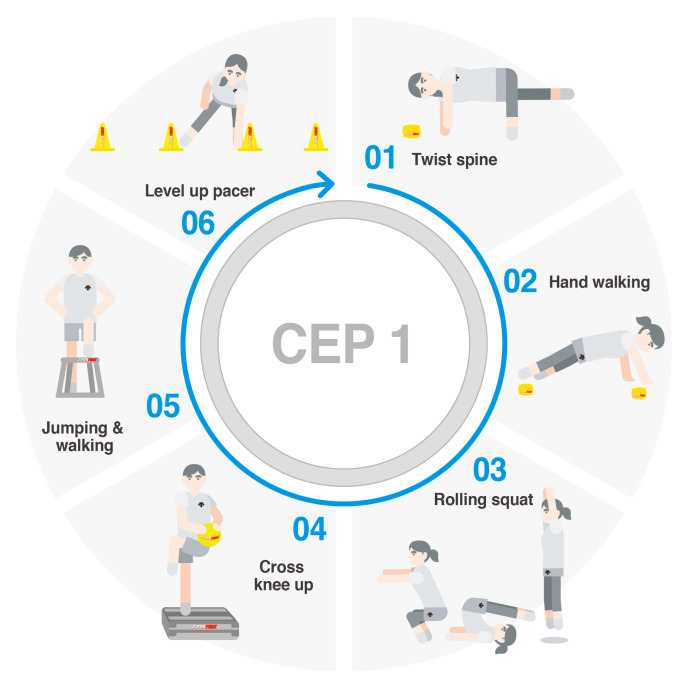
The sequence of motions for circuit exercise program 1: (1) Twist spine, (2) Hand walking, (3) Rolling squat, (4) Cross knee-up, (5) Jumping & walking, (6) Level-up pacer.

**Figure A2:**
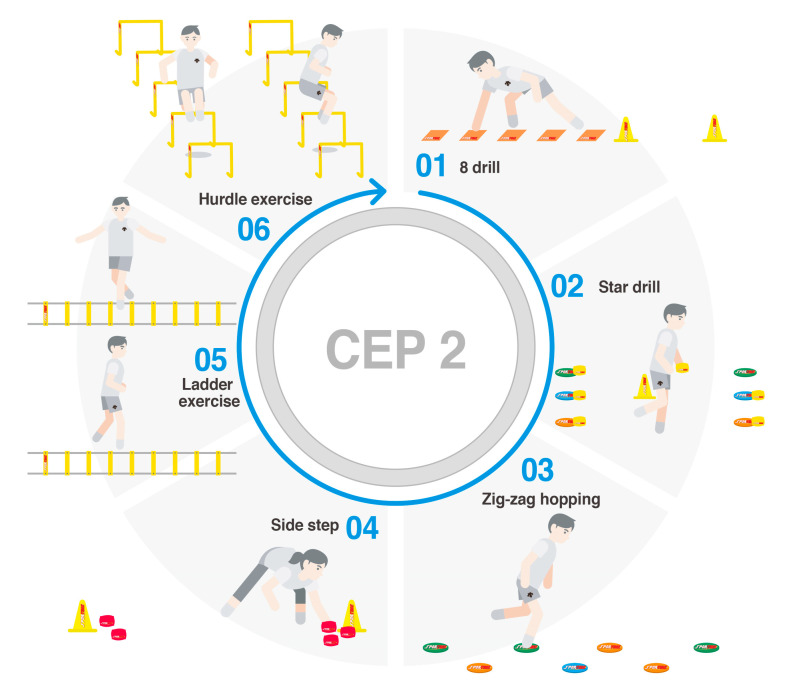
The sequence of motions for circuit exercise program 2: (1) 8 drill, (2) Star drill, (3) Zig-zag hopping, (4) Side step, (5) Ladder exercise, (6) Hurdle exercise.

**Figure A3:**
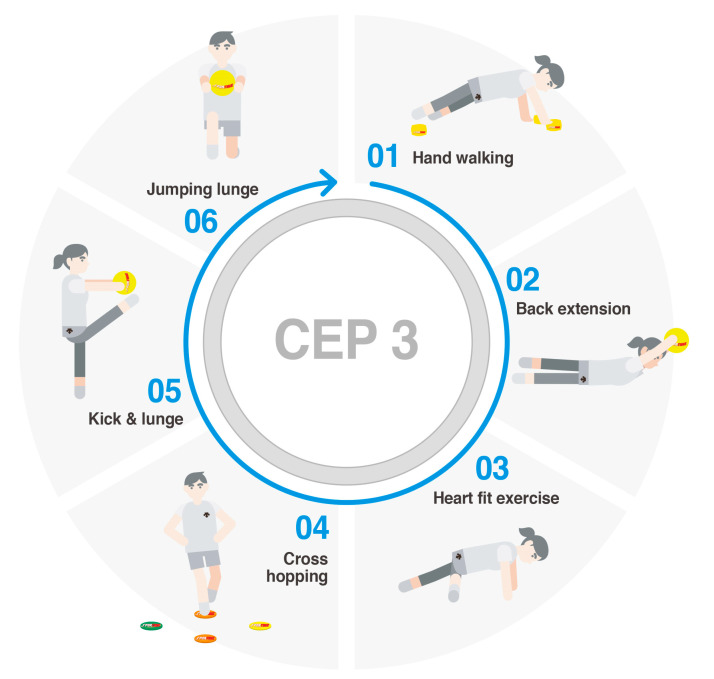
The sequence of motions for circuit exercise program 3: (1) Hand walking, (2) Back extension, (3) Heart fit exercise, (4) Cross hopping, (5) Kick & lunge, (6) Jumping lunge.

In order to present the criteria for circuit exercise programs used at school physical education sites, the Move Challenge developed by the health Physical Activity Institute was selected. The Move Challenge developed circuit exercise programs in the design, composition, and verification stages of physical education activities through the search process for related books and papers, and consultation with experts. Application and evaluation steps were carried out to verify effectiveness. The circuit exercise program in this study utilized adolescents’ physical activity promotion programs and consisted of fitness movements to promote health and fitness elements of endurance, flexibility strength, and power [13]. Subjects were measured for the data of the circuit exercise program in three separate physical education classes during the semester. The physical education classes had a washout period of one week, and the order of implementation of the circuit exercise program in each school was randomly selected. The circuit exercise program consists of six movements, as shown in Table 2 and Appendix A (Figure A1, Figure A2 and Figure A3). All movements were performed in sequence and recorded in seconds increments at the end of all movements.

### 2.3. Statics

The results from this study were analyzed using SPSS PC^+^ for Windows (version 20.0, IBM Corporation, Armonk, NY, USA). The following features were considered: (1) descriptive statistics of all variables are presented as mean (M) and standard deviation (SD); (2) one-way ANOVA was conducted to analyze the differences in the mean between dependent variables for each grade. If the mean difference in each grade is significant, then a post-hoc (Scheffe test) was conducted; (3) an independent *t*-test was conducted to analyze the mean differences in the dependent variables for gender. The significance level (a) for all statistical analyses is set to 0.05.

The five-level criteria of each circuit exercise program were divided into male and female students, and the percentile (%) was applied and presented. The five-level criteria were presented in separate phases, phase 1 (very high, 5% or less), phase 2 (high, 6% or more to less than 35%), phase 3 (normal, more than 35% to less than 65%), phase 4 (low, more than 65% to less than 95%), and phase 5 (very low, more than 95%).

## 3. Results

### 3.1. Comparison of Circuit Exercise Program by Grade and Gender

#### 3.1.1. Circuit Exercise Program 1

Table 3 shows the result of the comparison of circuit exercise program 1 by grade and gender. There were significant differences between grade in male (*p* = 0.000) and female (*p* = 0.000). Also, there were significant differences between male and female students in all grades except 5th grade.

#### 3.1.2. Circuit Exercise Program 2

Table 4 shows the result of the comparison of circuit exercise program 2 by grade and gender. There were significant differences between grade in male (*p* = 0.000) and female (*p* = 0.000). Also, there were significant differences between male and female students in all grades except the first grade.

#### 3.1.3. Circuit Exercise Program 3

Table 5 shows the result of the comparison of circuit exercise program 3 by grade and gender. There were significant differences between grade in male (*p* = 0.000) and female (*p* = 0.000). However, there were significant differences between second, third, and fifth grade, but there were no significant differences in first, fourth, and sixth grade.

### 3.2. Criteria of Circuit Exercise Program

**Figure 1:**
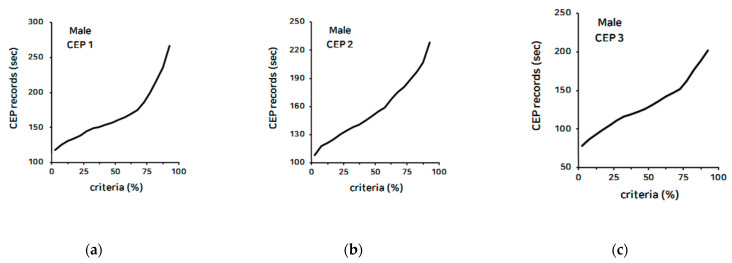
The criteria of circuit exercise program for males: (**a**) circuit exercise program 1; (**b**) circuit exercise program 2; (**c**) circuit exercise program 3.

**Figure 2:**
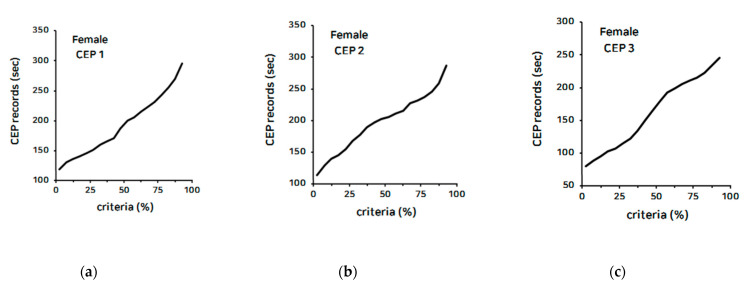
The criteria of circuit exercise program for females: (**a**) circuit exercise program 1; (**b**) circuit exercise program 2; (**c**) circuit exercise program 3.

Table 6 and Figure 1 and Figure 2, presents the criteria based on the results of each circuit exercise program obtained from the measurement. The results obtained from the measurement were presented on a five-step criteria, depending on the percentile.

## 4. Discussion

The decrease in physical activity of adolescent students has emerged as an educational, social, and national problem due to poor health and physical fitness. In order to prevent and improve obesity and physical fitness due to lack of physical activity in adolescents, regular physical activity should be conducted to maintain health and physical fitness factors at a certain level [18]. The Ministry of Education implements PAPS as a way to solve the health and physical fitness problems of adolescents. Various prior studies suggest methods for adolescent fitness assessments but lack the ability to improve health and physical fitness. In addition, criteria are insufficient in exercise programs developed to improve the health and fitness of adolescent students. This study utilized a circuit exercise program developed to improve adolescent health and physical fitness. Based on fitness movements, it can be carried out indoors, at home, and in narrow spaces without restrictions on the environment, so it can be highly utilized. A circuit exercise program was implemented for adolescents, and it was intended to present criteria for circuit exercise programs based on gender that can be used at physical education sites.

### 4.1. Comparison of Circuit Exercise Programs by Grade

In order to prevent obesity and deterioration of physical fitness among Korean adolescent students, the school’s physical education field is conducting PAPS. In addition, a comprehensive fitness assessment is conducted based on physical fitness for health assessments, and information on health and physical condition is provided [7]. Evaluating students’ health and physical fitness and conducting physical activities on low-level students is a very important role in school physical education. To improve student health and physical fitness levels, physical activities are encouraged, including health factors such as endurance, strength, and agility [19].

Although PAPS assesses student health and physical fitness levels, physical activity programs that improve students’ health and physical fitness levels at school physical education sites are lacking. In this study, the difference between grades was compared using circuit exercise programs that are effective in improving the health and physical fitness factors of adolescents. As a result of implementing the circuit exercise program, significant differences were shown by grade. A recent study comparing physical fitness to secondary school students showed that physical fitness improved as grades increased [19]. The results of this study were different from previous studies comparing the physical fitness of adolescents. In Korea, students are very interested in grades and college entrance, and there is a phenomenon of avoiding classes that do not help grades and entrance to college, especially as they move through grade levels and enter high school. In addition, students’ physical education classes are decreasing as the school grade level increases [9]. In this study, the influence of entering from middle school (first to third grade) to high school (fourth to sixth grade) seems to have resulted in different results from previous studies. However, in the same study, the lower the grade, the higher the physical fitness perception, supporting the findings of this study [20].

The circuit exercise program sequentially performs various forms of exercise. The circuit exercise program of the Move Challenge consists of self-practicable movements without restrictions of place and time constraints to prevent obesity and physical fitness deterioration in adolescents. Various physical activity programs for adolescents are known through previous studies, but the continuous and voluntary practice is difficult. In a study by Han Yeon-Oh et al. (2009), the physical self-concept and self-efficacy of psychological factors were improved, and the body mass index was effectively reduced through student health promotion programs [21]. Another study reported the improvement of psychological factors and physical factors, such as confidence in physical activity, physical activity volume, and body fat through adolescents’ physical activity programs [22]. In addition, adolescent physical activity habits have an important effect on adulthood physical activity habits and are closely related to health factors of life, recommended that MVPA be implemented to promote adolescents’ health and physical fitness [23]. Therefore, in order for adolescent students to improve physical and psychological factors, continuous physical activity is required.

In this study, the difference in physical fitness by grade was compared by conducting a circuit exercise program for adolescents. As a result of the adolescents’ circuit exercise program, there were significant differences by grade. Due to the nature of the circuit exercise program, various exercises can be experienced; however, it is difficult to evaluate the fitness level of each movement. Therefore, it is difficult to evaluate a specific physical fitness element, so an underestimation may appear depending on the degree of development of the physical fitness element. It is necessary to develop a method for measuring physical fitness factors according to each movement of the circuit exercise program.

### 4.2. Comparison of Circuit Exercise Programs by Gender

In this study, the results of adolescent circuit exercise programs differed according to gender. In a previous study, the physical fitness of adolescents growing up was compared according to their physique. It was reported that overweight and obese adolescents showed lower physical fitness than their normal-weight peers, and that a physique within the normal range was associated with high physical fitness. However, overweight and obese male adolescents showed higher fitness levels than female adolescents. These results were related to fat mass and free fat mass, and it was interpreted as the result that male adolescents had higher free fat mass than female adolescents [24]. In this study, male students showed a higher record of circuit exercise programs than female students. This is the same result as the previous study, and it is interpreted as the result of male students in the growing stage having higher lean body mass than female students.

In the results of this study, in the case of the first grade (14 years old), there was no difference in the records of circuit exercise programs between male adolescents and female adolescents. In a study by Ortega et al., there was no difference in physical fitness between male and female students. This was explained by the high homogeneity of the sample [25]. In the results of this study, there was no difference in the records of the circuit exercise program for the first graders, but a large difference appeared as the grades went up. In the case of the first grade, the development of female adolescents was high in elementary school, but when they entered middle school, the growth level of male adolescents was reversed. Differences according to gender in the circuit exercise program were similar to the results shown in previous studies. However, this study did not include the measurement of the adolescents’ physique level, so it was not possible to determine the gender difference according to the difference in the physique level.

The adolescent circuit exercise program has two purposes. (1) To improve physical fitness by satisfying the amount of physical activity through continuous implementation, and (2) to contribute to the presentation of goals for each fitness level by providing physical fitness evaluation standards. In future studies, it is considered necessary to investigate the effect of continuous adolescent circuit exercise programs on the physique and physical strength level of adolescents.

### 4.3. Development of Criteria for Circuit Exercise Program

Adolescents are at a time when physical factors such as physique and physical fitness develop, and the lack of physical activity appears to be a decrease in physical fitness. A decrease in physical fitness leads to a decrease in motivation for participation in physical activity. This process leads to a vicious cycle that negatively affects adolescents’ physical fitness for health [26]. Operational effects are insufficient due to the lack of understand of PAPS by ordinary teachers. Various methods of operation are required to improve student health and physical fitness [27]. Efforts are continuing to improve the environment of physical activities and develop programs and fitness evaluation tools to solve problems of decreasing adolescents’ health and physical fitness. In particular, it is important to set criteria for physical activity for adolescent students. High criteria do not create a sense of purpose, while low criteria make it difficult to induce motivation for accomplishment. Therefore, appropriate criteria for the purpose, target, and situation are required, along with physical activity programs that can improve adolescents’ physical fitness at school physical education sites [28].

PAPS assesses students’ health and physical fitness levels and prescribes appropriate physical activity [29]. School physical education provides physical activities to improve health and physical fitness for students who received low evaluation of PAPS. The absence of school physical education due to social and environmental problems such as fine dust and infectious appeared. In addition, the lack of physical activity programs that the opportunities for physical activity among adolescents are insufficient. In 2020, the Ministry of Education conducted a “Untact National school sports Club Festival” for adolescents whose physical activity opportunities were reduced due to coronavirus [30]. For the purpose of invigoration, the school sports club, elementary, middle and high school students across the country were given the opportunity to participate in 15 sports events. Furthermore, the Descente Sports Foundation conducted the ‘2020 Online Move Challenge’ [31,32]. Move Challenge was developed by the Health Physical Activity Institute as a circuit exercise program consisting of six movements and supported the physical fitness activities of middle and high school students [13,31]. Through these Untact events, it was suggested that criteria for physical activity programs are needed so that adolescents can continue to participate in physical activities and voluntarily practice health and physical fitness management suitable for individual levels.

As students go up in grade, voluntary health and physical activities decrease. It was suggested that voluntary participation should be induced in a variety of ways to improve the health habits of adolescents [33]. A previous study suggested that improvement in the method of measuring and managing students’ physical fitness was necessary and reported that the utilization of circuit exercise programs will be high at school physical education sites. In addition, the Move Challenge was correlated with the PAPS measurement results, suggesting the possibility of a physical fitness measurement method [34]. In this study, set criteria with adolescents’ circuit exercise programs provided by the Health Physical Activity Institute were presented. Each circuit exercise program showed significant differences in grade and gender. 

It may be necessary to present evaluation criteria for each grade in consideration of the impact on the development, and growth of students. However, in order to provide convenience at school physical education sites, the evaluation criteria for the circuit exercise program were presented in five steps, depending on gender. The circuit exercise programs were difficult to compare the effects of each fitness factor and movement. However, it was reported positively to improve students’ overall physical strength due to the high utilization at school physical education sites [12,35]. The circuit exercise program of adolescent students conducted in this study is believed to be used as a physical activity program to set goals for adolescent students. The circuit exercise program conducted in this study can be used as a physical activity program for adolescent students to establish their physical activity goals. In the future, it is necessary to study the comparison of physical fitness of adolescents using circuit exercise programs and establish criteria for various adolescents’ physical activity programs.

## 5. Conclusions

This study intended to propose criteria based on the results of a circuit exercise program developed to improve the health and physical fitness of adolescents students. As a result, both grade and gender differences were shown in each of the developed circuit exercise program. In order to enhance the utilization and convenience of various adolescents’ physical activities environments and school physical education sites, five-step criteria were presented according to gender. The circuit exercise program of this study will be utilized for various adolescents’ physical activities to contribute to the improvement of the health and physical fitness levels of adolescent students. In addition, it is believed that it provides convenience for teachers by being used as a criterion for briefly evaluating students’ physical fitness levels in various adolescent physical activity environments.

## Figures and Tables

**Table 1:** Sample size by gender and grade.

Gender	Grade	Years	CEP 1 (*n* = 1719)	CEP 2 (*n* =1830)	CEP 3 (*n* =1067)	BMI (kg·m^−2^)
Male	1st grade	14 years (*n* = 493)	284	117	92	22.2 ± 11.90
	2nd grade	15 years (*n* = 399)	149	172	78	21.8 ± 4.58
	3rd grade	16 years (*n* = 456)	185	200	71	22.7 ± 4.69
	4th grade	17 years (*n* = 592)	161	240	191	23.1 ± 4.71
	5th grade	18 years (*n* = 827)	199	382	246	22.4 ± 3.92
	6th grade	19 years (*n* = 325)	100	107	108	23.9 ± 4.06
Female	1st grade	14 years (*n* = 411)	241	108	62	20.4 ± 3.90
	2nd grade	15 years (*n* = 378)	163	174	41	20.2 ± 3.78
	3rd grade	16 years (*n* = 232)	98	93	41	22.0 ± 3.47
	4th grade	17 years (*n* = 111)	35	41	35	20.5 ± 2.37
	5th grade	18 years (*n* = 308)	72	164	72	20.9 ± 3.14
	6th grade	19 years (*n* = 94)	32	32	30	20.4 ± 2.32

CEP: circuit exercise program, BMI: body mass index.

**Table 2:** Methods of circuit exercise program.

Program	Item	Contents
CEP 1	Twist spine (flexibility)	put one leg over the pelvis, fix it on the floor, and then move one bean bag while turning the upper body to the other side (2 sets of 3 repetitions for each side left and right).
	Hand walking (strength)	in the push-up posture, move one bean bag by hand to the tip of the toe in order and then return to the place (1 set of 2 repetitions).
	Rolling squat (strength)	prepare in a squat position, roll back until toes are over your head, and roll forward to squat position (1 set of 5 repetitions).
	Cross knee up (endurance)	(1) cross-jump on the step box (10 repetitions) while holding the ball. (2) bend your knees and cross the ball diagonally (5 repetitions on each side alternately). (3) extend your knees and cross the ball diagonally (5 repetitions on each side alternately).
	Jumping & walking (power)	(1) jump on top of the box with both feet (5 repetitions). (2) step up the box (5 repetitions).
	Level-up pacer (endurance)	shuttle run by touching the four cones standing at 1.5 m intervals in sequence.
CEP 2	Eight-drill (agility)	run in an 8-shape shape and touch the target points in order (1 set).
	Star drill (agility)	(1) move quickly from center point to target point. (2) move 1 bean bag to each target point (1 set).
	Zig-zag hopping (power)	(1) jump on both feet and move along the maker (1 set). (2) jump on one foot and move along the maker (once each left and right).
	Side-step (agility)	move one bean bag to the target point while moving to the side step (1 set of 5 repetitions).
	Ladder exercise (agility)	(1) move the side of the ladder to the front step (1set). (2) move the side of the ladder to the side step (1 set). (3) move the ladder head-on in the cross-step (1 set). (4) move the ladder sideways in the cross-step (1 set).
	Hurdle exercise (agility)	(1) jump on both feet and cross the hurdle (1 set). (2) jump on both feet and cross the hurdle sideways (once each left and right).
CEP 3	Hand walking (strength)	In the push-up posture, move one bean bag by hand to the tip of the toe in order and then return to the place (1 set of 2 repetitions).
	Back extension (strength)	(1) hold the ball with both hands and lift the upper body (5 repetitions). (2) place the ball between both legs and lift the lower body (5 repetitions). (3) lift the upper and lower body at the same time (5 repetitions).
	Heart fit exercise (endurance)	(1) place both hands on the floor, move forward, lie down, fold and straighten your legs. (2) lift your knees off the floor and stretch your upper body back. (3) in the push-up position, kick one leg up to shoulder level (once each left and right). (4) in the push-up position, place one hand on the floor and lift the other arm up so that it is perpendicular to the floor (once each left and right).(5) in the push-up position, as you jump, you widen your feet to the side, and as you jump, you gather both feet (5 repetitions). (6) in the push-up position, put your feet together, jump 3 times towards your upper body, and jump 3 times to get back into place (2 repetitions). (7) in the push-up position, move both hands toward the body and stand straight.
	Cross hopping (power)	(1) move the markers in order by hopping (front-back-left-right, 1 set each left and right). (2) move the markers in order by hopping (once each clockwise and counterclockwise).
	Kick & lunge (strength)	after lunge, stretch your back legs forward and touch the ball with your toes (left and right each 5 repetitions)
	Jumping lunge (power)	(1) alternately the feet and move forward by jumping & lunge (5 repetitions). (2) alternately the feet and move forward by jumping & lunge (5 repetitions).

CEP: circuit exercise program, motion images of circuit exercise programs, CEP 1: https://youtu.be/ht5T4qWSb84 (accessed on 24 June 2019) [15], CEP 2: https://youtu.be/Vdqyk5nuCc8 (accessed on 24 June 2019) [16], CEP 3: https://youtu.be/FS9pP4TaZvc (accessed on 24 June 2019) [17].

**Table 3:** Comparison of circuit exercise program 1 by grade and gender (Mean ± SD).

Program	Gender	1st Grade	2nd Grade	3rd Grade	4th Grade	5th Grade	6th Grade	*F*-Value	*p*-Value	Post-Hoc
CEP 1	Male (*n* = 1078)	119.3 ± 38.66 *	155.1 ± 27.99 ***	187.7 ± 66.28 *	174.6 ± 42.02 **	164.9 ± 37.65	183.1 ± 50.97 ***	69.848	0.000	a < b, c, d, e, f b < c, d, f c > e
	Female (*n* = 641)	113.1 ± 33.85	200.6 ± 55.14	218.3 ± 74.55	209.3 ± 21.35	185.4 ± 31.80	207.8 ± 21.43	115.225	0.000	a < b, c, d, e, f c > e

CEP: circuit exercise program, a: grade 1, b: grade 2, c: grade 3, d: grade 4, e: grade 5, f: grade 6, * significant differences between gender, *: *p* < 0.05, **: *p* < 0.01, ***: *p* < 0.001.

**Table 4:** Comparison of circuit exercise program 1 by grade and gender (Mean ± SD).

Program	Gender	1st Grade	2nd Grade	3rd Grade	4th Grade	5th Grade	6th Grade	*F*-Value	*p*-Value	Post-Hoc
CEP 2	Male (*n* = 1218)	188.1 ± 42.83	156.7 ± 38.71 ***	155.4 ± 29.67 ***	163.6 ± 47.99 ***	152.4 ± 33.66 ***	164.3 ± 42.52 **	16.773	0.000	a > b, c, d, e, f d > e
	Female (*n* = 612)	194.6 ± 45.82	214.5 ± 59.74	189.8 ± 52.61	200.5 ± 27.30	183.9 ± 47.73	206.5 ± 26.14	7.200	0.000	b > c, e

CEP: circuit exercise program, a: grade 1, b: grade 2, c: grade 3, d: grade 4, e: grade 5, f: grade 6, * significant differences between gender, **: *p* < 0.01, ***: *p* < 0.001.

**Table 5:** Comparison of circuit exercise program by grade and gender (*Mean ± SD*).

Program	Gender	1st Grade	2nd Grade	3rd Grade	4th Grade	5th Grade	6th Grade	*F*-Value	*p*-Value	Post-Hoc
CEP 3	Male (*n* = 786)	120.1 ± 50.91	109.0 ± 26.89 ***	101.1 ± 27.10 ***	143.7 ± 36.48	145.3 ± 36.17 ***	131.6 ± 28.02	29.931	0.000	a > d, e b < d, e, f c < a, d, e, f
	Female (*n* = 281)	123.2 ± 57.11	132.9 ± 58.97	125.1 ± 53.90	202.1 ± 41.46	175.0 ± 42.77	216.0 ± 23.27	27.994	0.000	a < d, e, f b > d, e, f c < d, e, f e < f

CEP: circuit exercise program, a: grade 1, b: grade 2, c: grade 3, d: grade 4, e: grade 5, f: grade 6, ***: *p* < 0.001.

**Table 6:** Criteria of circuit exercise program (Unit: sec).

Program	Gender	Phase 5 (Very Low)	Phase 4 (Low)	Phase 3 (Normal)	Phase 2 (High)	Phase 1 (Very High)
CEP 1	Male	266 or higher	265~170	169~149	148~119	118 or less
	Female	295 or higher	294~215	214~160	159~120	119 or less
CEP 2	Male	228 or higher	227~168	167~138	137~109	108 or less
	Female	287 or higher	246~215	214~178	177~115	114 or less
CEP 3	Male	202 or higher	201~142	141~116	115~79	78 or less
	Female	245 or higher	244~199	198~122	121~81	80 or less

CEP: circuit exercise program.

## Data Availability

The data presented in this study are available on request from the corresponding author.
